# High aldehyde dehydrogenase activity identifies cancer stem cells in human cervical cancer

**DOI:** 10.18632/oncotarget.1578

**Published:** 2013-11-25

**Authors:** Shu-Yan Liu, Peng-Sheng Zheng

**Affiliations:** ^1^ Department of Reproductive Medicine, The First Affiliated Hospital of the Medical College, Xi'an Jiaotong University, Xi'an, The People's Republic of China; ^2^ Section of Cancer Stem Cell Research, Key Laboratory of Environment and Genes Related to Diseases, Ministry of Education of the People's Republic of China, Xi'an, The People's Republic of China

**Keywords:** aldehyde dehydrogenase, cancer stem cells, cervical cancer, self-renewal, chemoresistance

## Abstract

High aldehyde dehydrogenase (ALDH) activity characterizes a subpopulation of cells with cancer stem cell (CSC) properties in several malignancies. To clarify whether ALDH can be used as a marker of cervical cancer stem cells (CCSCs), ALDH^high^ and ALDH^low^ cells were sorted from 4 cervical cancer cell lines and 5 primary tumor xenografts and examined for CSC characteristics. Here, we demonstrate that cervical cancer cells with high ALDH activity fulfill the functional criteria for CSCs: (1) ALDH^high^ cells, unlike ALDH^low^ cells, are highly tumorigenic *in vivo*; (2) ALDH^high^ cells can give rise to both ALDH^high^ and ALDH^low^ cells *in vitro* and i*n vivo*, thereby establishing a cellular hierarchy; and (3) ALDH^high^ cells have enhanced self-renewal and differentiation potentials. Additionally, ALDH^high^ cervical cancer cells are more resistant to cisplatin treatment than ALDH^low^ cells. Finally, expression of the stem cell self-renewal-associated transcription factors OCT4, NANOG, KLF4 and BMI1 is elevated in ALDH^high^ cervical cancer cells. Taken together, our data indicated that high ALDH activity may represent both a functional marker for CCSCs and a target for novel cervical cancer therapies.

## INTRODUCTION

Cervical cancer is the second most commonly diagnosed cancer and ranks second only to breast cancer as the leading cause of cancer death in women in developing countries [1]. Based on the GLOBOCAN estimates, approximately 529,000 women worldwide were diagnosed with invasive cervical cancer, and more than half of these patients died from their disease in 2008 [2]. Cervical carcinoma development begins with the infection of the cervical epithelium by high-risk human papillomaviruses (hr-HPVs) [3-5]. Although cervical cancer can be detected in its early stages by HPV testing and Papanicolaou (Pap) smear screening and successfully eradicated through surgery, curative treatments do not yet exist for advanced, recurrent or metastatic disease [6-8].

Tumor growth and metastasis are driven by a small population of cancer stem cells (CSCs) [9]. The first extensive documentation of CSCs came from leukemia, in which only a small subset of cancer cells were shown to be capable of transferring the disease to non-obese diabetic/severe combined immunodeficient (NOD/SCID) mice [10]. This concept was then extended to solid tumors. Human breast cancers have been demonstrated to contain a population of cells with stem cell properties that display surface marker expression of CD44^+^/CD24^−^/lin^−^ [11]. Subsequently, CSCs have been identified and prospectively isolated using surface markers from a variety of malignancies, including brain tumors [12], melanoma [13], multiple myeloma [14], prostate cancer [15], colon cancer [16, 17], head and neck squamous cell carcinoma [18, 19] and pancreatic adenocarcinoma [20]. Due to the instability and scarcity of surface markers in solid tumors, other methodological strategies have been widely explored to identify and isolate CSCs, including side population phenotype, sphere formation and aldehyde dehydrogenase (ALDH) activity assay [21].

ALDH is a group of intracellular enzymes that oxidize aldehydes (thereby serving a detoxifying role) and convert retinol to retinoic acid, which mediates control over differentiation pathways [22, 23]. ALDH is highly expressed in hematopoietic stem cells and provides protection against the alkylating agent cyclophosphamide [24-27]. Increasing evidence has suggested that ALDH activity can be used either alone or in combination with cell surface markers to identify CSCs in hematologic malignancies [28] and a steadily increasing number of solid tumors, including those of the breast [29], colon [30-32], bladder [33], prostate [34, 35], lung [36], pancreas [37], head and neck [38], endometrium [39], ovary [40] and melanoma [41]. Previous studies have shown that ALDH1 is expressed in cervical cancer cell lines and primary cervical cancer tissues [42-44]. However, the ability to isolate and identify cervical cancer stem cells (CCSCs) by ALDH activity has not yet been reported.

To verify whether cells with high ALDH activity are CCSCs, fluorescence activated cell sorting (FACS) and standard functional assays were used in the present study to analyze the cellular properties of ALDH^high^ and ALDH^low^ cells isolated from 4 human cervical cancer cell lines and 5 primary cervical cancers. The results indicated that a subpopulation of human cervical cancer cells with high ALDH activity possess enhanced self-renewal capacities, differentiation potential and increased tumorigenicity, indicating that high ALDH activity may represent a marker of CSCs in cervical cancer.

## RESULTS

### ALDH expression and activity in human cervical tissue specimens and cervical cancer cell lines

**Figure 1:**
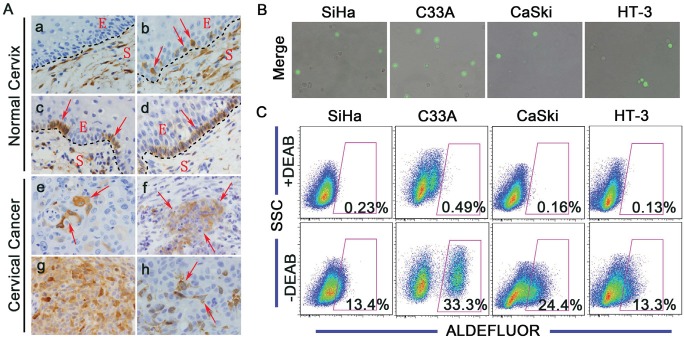
ALDH expression in human cervical tissue specimens and cervical cancer cell lines A, Representative photos of immunostained specimens showing ALDH1 expression in normal cervical (a-d) and cervical cancer (e-h) tissues. a, no ALDH1-positive cells; b, dot-scattered ALDH1 expression; c, focal distribution of ALDH1-positive cells; d, all basal cells of the normal cervix are ALDH1-positive; e, dot-scattered ALDH1 expression; f, focal distribution of ALDH1-positive cells; g, diffuse ALDH1 expression; h, ALDH1-positive cells in xenograft tissue from mice. Red arrows indicate ALDH1-positive cells. S, Stroma; E, Epithelium; Magnifications, 1000×. B, Cells were labeled using the ALDEFLUOR kit, and ALDH^high^ cells (bright green fluorescence) were detected by fluorescent microscopy. C, ALDH enzyme activity in 4 cervical cancer cell lines was analyzed by flow cytometry. As a negative control, cells were treated with the specific ALDH inhibitor DEAB. The gated cells represent the ALDH^high^ cells.

ALDH1 expression was evaluated in normal and cancerous cervical tissues (Figure 1A). Notable differences were observed in the expression patterns of ALDH1 in the basal cells of normal human cervical tissues; these patterns were classified into 4 types: (1) No ALDH1 expression (Figure 1Aa); (2) Dot-scattered ALDH1 expression (Figure 1Ab); (3) Focal distribution of ALDH1-positive cells (Figure 1Ac); and (4) Positive ALDH1 expression in all basal cells (Figure 1Ad). Because basal cells are known to contain undifferentiated reserve cells of the normal cervix, we speculated that the ALDH1-positive basal cells may represent the stem cells of the normal cervix. A large number of ALDH1-positive cells were found in the stroma in all of the normal cervical tissues (Figure 1Aa-1Ad). Most of these ALDH1-positive cells in the normal cervical stroma are likely CD45-positive leukocytes, which have also been found in the stroma of normal breast tissues by Dr. Deng et al. [45].

ALDH1-positive cells were also found in all 53 cervical cancer tissues. Similar to ALDH1 expression in the normal cervical tissues, the expression patterns of ALDH1 in the cervical cancer tissues could be classified into 3 types: (1) Dot-scattered ALDH1 expression (Figure 1Ae); (2) Focal distribution of ALDH1-positive tumor cells (Figure 1Af); and (3) Diffuse ALDH1 expression (Figure 1Ag). In this study, approximately 10% of the cervical cancer cells were ALDH1-positive, which is consistent with the notion that CSCs constitute a minority of the tumor cells. Furthermore, ALDH1-positive cells were present in each of the 19 passages of the serially xenografted tissues in NOD/SCID mice (Figure 1Ah).

The ALDEFLUOR kit was used to test the ALDH enzymatic activity in the cervical cancer cell lines. Cells were labeled with activated ALDEFLUOR reagent in the presence or absence of the ALDH inhibitor, DEAB. A drop of ALDEFLUOR-labeled cells was smeared and examined by fluorescence microscopy. Fluorescent and phase contrast images were acquired and merged. As shown in Figure 1B, each of the 4 cervical cancer cell lines (SiHa, C33A, CaSki and HT-3) contained ALDH-positive cells (indicated by the bright green fluorescence). The remaining ALDEFLUOR-labeled cells were analyzed by flow cytometry. Compared with the DEAB-treated control, high ALDH activity was detected in 13.4% of the SiHa cells, 33.3% of the C33A cells, 24.4% of the CaSki cells and 13.3% of the HT-3 cells (Figure 1C).

Together, these results suggest that a subpopulation of ALDH^high^ cells exists in normal and cancerous cervical tissues, serially xenografted cervical cancer tissues and cervical cancer cell lines, implying that ALDH may be a marker of stem cells and CSCs in cervical tissues.

### ALDH^high^ cervical cancer cells display enhanced self-renewal capacity

**Figure 2:**
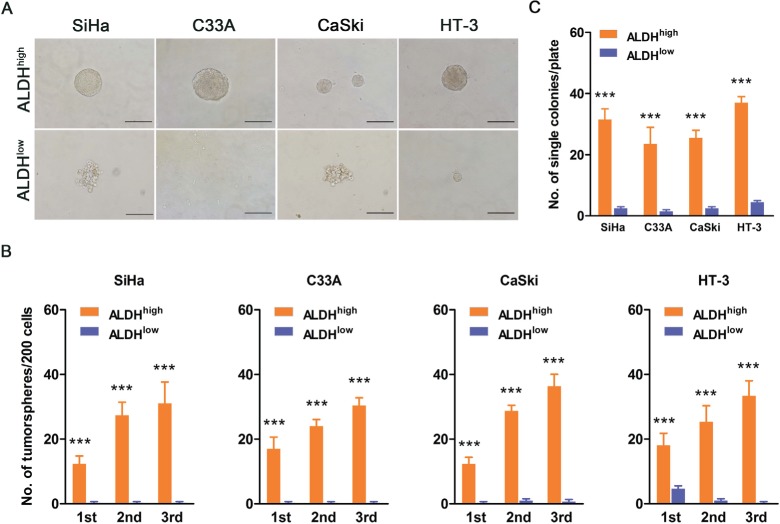
ALDH^high^ cervical cancer cells exhibit enhanced self-renewal capacity A, Representative photos of tumorspheres formed by ALDH^high^ and ALDH^low^ cells are shown. Bar, 200 µm. B, The number of tumorspheres/200 cells was counted from 3 consecutive passages. C, The number of wells containing tumorspheres was counted. ^***^, *p*<0.001. Data represent mean ± SD of triplicate experiments.

Self-renewal is a critical characteristic of stem cells and CSCs. To assess self-renewal *in vitro*, ALDH^high^ and ALDH^low^ cervical cancer cells were cultured in serum-free medium under conditions optimal for growing tumorspheres. As shown in Figure 2A, ALDH^high^ cells isolated from the 4 cervical cancer cell lines generated classical tumorspheres, while ALDH^low^ cells did not form tumorspheres, but only a few cell aggregates. When plated at a density of 200 cells/well in 24-well plates (low density culture), 6.2%, 8.5%, 6.2% and 9% of the ALDH^high^ cells from SiHa, C33A, CaSki and HT-3 cells, respectively, generated tumorspheres in the 1^st^ passage, while the ALDH^low^ cells generated no, or very rare, tumorspheres. Upon 3 consecutive passages in culture, the tumorsphere forming efficiency of the ALDH^high^ cells gradually increased (Figure 2B). To exclude the effects of cell aggregation, which can occur in low density cultures, cells were cultured at a density of a single cell/well. The ALDH^high^ cells from SiHa, C33A, CaSki and HT-3 cells generated tumorspheres with an efficiency of 32.8%, 24.5%, 26.6% and 38.5%, whereas the ALDH^low^ cells generated tumorspheres with an efficiency of 2.6%, 1.6%, 2.6% and 4.7%, respectively (Figure 2C). These data indicated that the ALDH^high^ cervical cancer cells have greater self-renewal capacity than the ALDH^low^ cells.

### ALDH^high^ cervical cancer cells have enhanced tumorigenic capacity *in vivo*

One of the most important characteristics of CSCs is their powerful ability to form tumors. To determine whether the ALDH^high^ cells have a greater capacity to form tumors, the ALDH^high^ and ALDH^low^ cell populations were sorted from 4 cervical cancer cell lines, and limiting dilutions of the cells were subcutaneously injected into NOD/SCID mice. The tumor latency, tumor incidence and tumor volume were then monitored.

**Figure 3:**
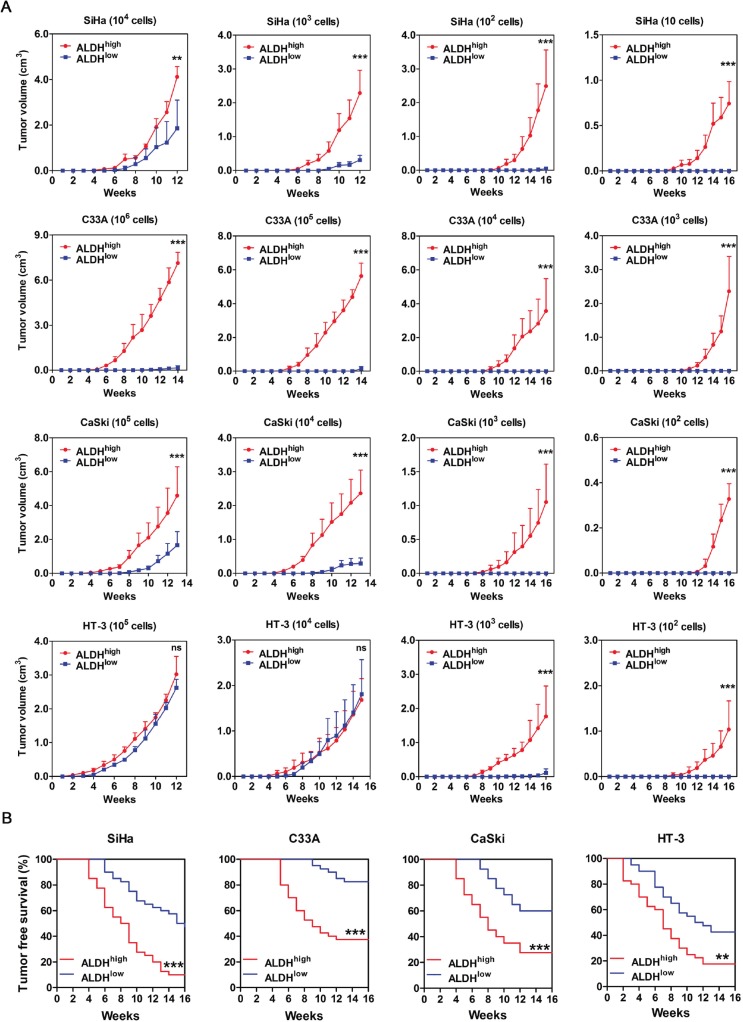
Tumorigencity of ALDH^high^ and ALDH^low^ cells from 4 cervical cancer cell lines in NOD/SCID mice A, The volume of xenograft tumors formed by different numbers of ALDH^high^ and ALDH^low^ cervical cancer cells was monitored over time. B, Kaplan-Meier plots showing the tumor-free survival after injection. ns, not significant; ^*^, *p*<0.05; ^**^, *p*<0.01; ^***^, *p*<0.001. Data represent mean ± SD of tumor volumes at different time points of 10 mice in each group.

Firstly, tumor volume was monitored twice a week, and the results are shown in Figure 3A. Inoculation of NOD/SCID mice with 10^4^ or 10^3^ ALDH^high^ and ALDH^low^ SiHa cells led to tumor formation from both populations. However, the tumors formed by ALDH^high^ SiHa cells were larger and grew faster than those formed by ALDH^low^ SiHa cells. Furthermore, inoculation with 10^2^ or 10^1^ ALDH^high^ and ALDH^low^ SiHa cells led to tumor formation from only the ALDH^high^ SiHa cells (Figure 3A, panel 1). In C33A cells, the ALDH^high^ population, but not the ALDH^low^ population, was capable of forming palpable tumors at each cell dose (10^6^, 10^5^, 10^4^ and 10^3^). However, upon sacrifice of the NOD/SCID mice, very small regions of tumor were found in the mice that had been inoculated with 10^6^ and 10^5^ ALDH^low^ C33A cells (Figure 3A, panel 2). Upon inoculation with 10^5^ or 10^4^ CaSki cells, the ALDH^high^ population formed larger palpable tumors more rapidly than the ALDH^low^ population. However, after inoculation with 10^3^ or 10^2^ cells, the ALDH^high^ CaSki cells, but not the ALDH^low^ CaSki cells, were capable of forming palpable tumors (Figure 3A, panel 3). In HT-3 cells, 10^5^ or 10^4^ of the ALDH^high^ and ALDH^low^ cells formed similarly sized palpable tumors almost simultaneously. When the cell dose was decreased to 10^3^ or 10^2^, ALDH^high^ HT-3 cells could form palpable tumors, while ALDH^low^ HT-3 cells formed very small or no palpable tumors (Figure 3A, panel 4).

Tumor latency was monitored after injection of sorted cells into the NOD/SCID mice and was defined by the period of time during which the mice remained tumor-free (Figure 3B). ALDH^high^ SiHa cells exhibited a significantly shorter tumor-free period; for instance, the shortest tumor-free period for ALDH^high^ cells was 4 weeks, as compared to the 6 week latent period for ALDH^low^ cells. ALDH^high^ SiHa cells also exhibited a lower tumor-free rate (10% in ALDH^high^ cells versus 47.5% in ALDH^low^ cells) than ALDH^low^ SiHa cells (*p*<0.001). Similarly, ALDH^high^ C33A cells displayed a significantly shorter tumor-free period (5 weeks in ALDH^high^ cells versus 9 weeks in ALDH^low^ cells) and a lower tumor-free rate (37.5% in ALDH^high^ cells versus 82.5% in ALDH^low^ cells) compared with ALDH^low^ C33A cells (*p*<0.001). ALDH^high^ CaSki cells exhibited a significantly shorter tumor-free period (4 weeks in ALDH^high^ cells versus 7 weeks in ALDH^low^ cells) and a lower tumor-free rate (27.5% in ALDH^high^ cells versus 60% in ALDH^low^ cells) compared with ALDH^low^ CaSki cells (*p*<0.001). ALDH^high^ HT-3 cells also showed a significantly shorter tumor-free period (2 weeks in ALDH^high^ cells versus 3 weeks in ALDH^low^ cells) and a lower tumor-free rate (17.5% in ALDH^high^ cells versus 42.5% in ALDH^low^ cells) compared with ALDH^low^ HT-3 cells (*p*<0.01).

The tumor incidence from ALDH^high^ and ALDH^low^ populations in all 4 cervical cancer cell lines is summarized in Table 1. The tumor-initiating frequency of ALDH^high^ SiHa cells was 1/11, which was 51.4-fold higher than that of ALDH^low^ SiHa cells (1/586; *p*<0.001). The tumor-initiating frequency of ALDH^high^ C33A cells (1:27,331) was 39.8–fold higher than that of ALDH^low^ C33A cells (1:1,086,487; *p*<0.001). The tumor-initiating frequency of ALDH^high^ CaSki cells was 1/737, which was 17.4-fold higher than that of ALDH^low^ CaSki cells (1/12,788; *p*<0.001), and the tumor-initiating frequency of ALDH^high^ HT-3 cells (1:234) was 14.7-fold higher than that of ALDH^low^ HT-3 cells (1:3,434; *p*<0.001).

**Table 1:** Tumorigenic capacity of ALDH^high^ and ALDH^low^ cells in NOD/SCID mice from 4 cervical cancer cell lines

Cell line	Sub-population	Cell dose						Tumor-Initiating frequency (95% Interval)	p value
		10^6^	10^5^	10^4^	10^3^	10^2^	10		
SiHa	ALDH^high^	--	--	10/10	10/10	10/10	6/10	1:11(1:25—1:5)	<0.001
	ALDH^low^	--	--	10/10	7/10	4/10	0/10	1:586(1:1,162—1:295)	
C33A	ALDH^high^	10/10	9/10	4/10	2/10	--	--	1:27,331(1: 57,546—1:12,981)	<0.001
	ALDH^low^	6/10	1/10	0/10	0/10	--	--	1:1,086,487(1:2,331,530—1:506,300)	
CaSki	ALDH^high^	--	10/10	10/10	7/10	2/10	--	1:737(1:1,496—1:363)	<0.001
	ALDH^low^	--	10/10	6/10	0/10	0/10	--	1:12,788(1:28,040—1:5,833)	
HT-3	ALDH^high^	--	10/10	10/10	10/10	3/10	--	1:234(1:520—1:105)	<0.001
	ALDH^low^	--	10/10	9/10	4/10	0/10	--	1:3,434(1:6,968—1:1,692)	

Together, these results from the tumor formation assays in NOD/SCID mice suggest that ALDH^high^ cervical cancer cells have a more rapid tumor growth rate, shorter tumor latency, lower tumor-free rate and higher tumor-initiating frequency than ALDH^low^ cells. Therefore, ALDH^high^ cervical cancer cells have a potent ability to form tumors *in vivo*.

### ALDH^high^ cells, but not ALDH^low^ cells, have the ability to differentiate *in vitro* and *in vivo*

**Figure 4:**
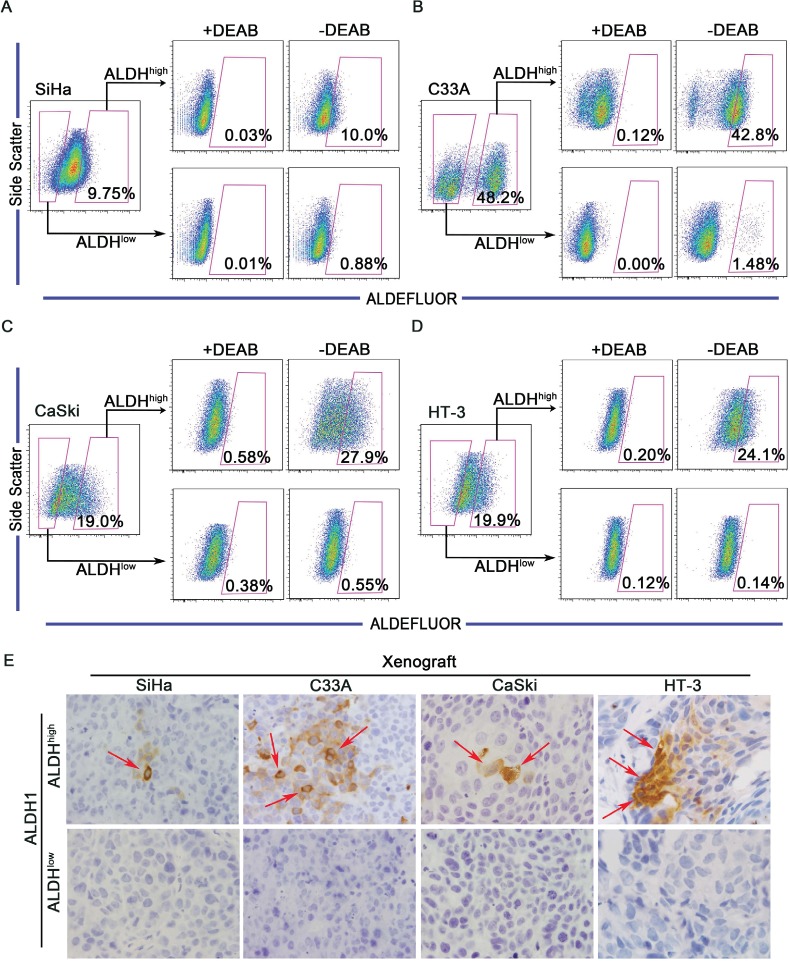
ALDH^high^ cervical cancer cells are capable of differentiating ^in vitro^ and ^in vivo^ A-D, ALDH^high^ and ALDH^low^ cells were isolated from the SiHa (A), C33A (B), CaSki (C) or HT-3 (D) cell lines and cultured in DMEM medium supplemented with 10% FBS for 2 weeks. The ALDH enzyme activity was then analyzed by flow cytometry. Cells treated with DEAB served as a negative control. The gated cells represent the ALDH^high^ cells. E, Expression of ALDH1 was detected by IHC in xenograft tumors from ALDH^high^ and ALDH^low^ cells. Red arrows indicate ALDH1-positive cells

One characteristic of CSCs is the capacity to differentiate into non-CSCs and give rise to the heterogeneous tumor cell populations. To determine whether ALDH^high^ cells are capable of differentiation *in vitro*, ALDH^high^ and ALDH^low^ cells were cultured separately in DMEM medium supplemented with 10% FBS for 2 weeks. After incubation, the cultured populations were analyzed using the ALDEFLUOR assay (Figure 4A-4D). Approximately 90% of the ALDH^high^ SiHa cells differentiated into ALDH^low^ cells, and only 10% of the cells remained strongly ALDH-positive. However, greater than 99% of the ALDH^low^ SiHa cells retained the ALDH^low^ phenotype (Figure 4A). Similarly, 57.2% of the ALDH^high^ C33A cells (Figure 4B), 72.1% of the ALDH^high^ CaSki cells (Figure 4C) and 75.9% of the ALDH^high^ HT-3cells (Figure 4D) generated ALDH^low^ cells. However, 98.52% of the ALDH^low^ C33A cells (Figure 4B), 99.45% of the ALDH^low^ CaSki cells (Figure 4C) and 99.86% of the ALDH^low^ HT-3 cells (Figure 4D) maintained the ALDH^low^ phenotype. Notably, 1.48% of the ALDH^low^ C33A cells generated ALDH^high^ cells, which was a larger fraction than the other cell lines. Because C33A cells contain a larger population of ALDH^high^ cells and higher ALDH activity than the other cell lines, this proportion of ALDH^high^ cells may have resulted from errors during the cell sorting manipulation.

The differentiation capacity of ALDH^high^ and ALDH^low^ cells was also assessed *in vivo*. In the tumors formed by ALDH^high^ cells, a few ALDH1-positive cells and many ALDH1-negative cells were found, indicating that ALDH^high^ cells were able to generate ALDH^high^ cells through self-renewal and to generate ALDH^low^ cells through differentiation (Figure 4E, upper panel). However, in the tumors formed by ALDH^low^ cells, no ALDH1-positive cells were found, indicating that ALDH^low^ cells did not have the ability to differentiate (Figure 4E, lower panel).

Taken together, these data demonstrate that ALDH^high^ cervical cancer cells have the ability to differentiate both *in vitro* and *in vivo*. Thus, ALDH^high^ cervical cancer cells establish the cellular hierarchy in tumors through self-renewal and differentiation.

### ALDH^high^ cells are more resistant to cisplatin than ALDH^low^ cells

**Figure 5:**
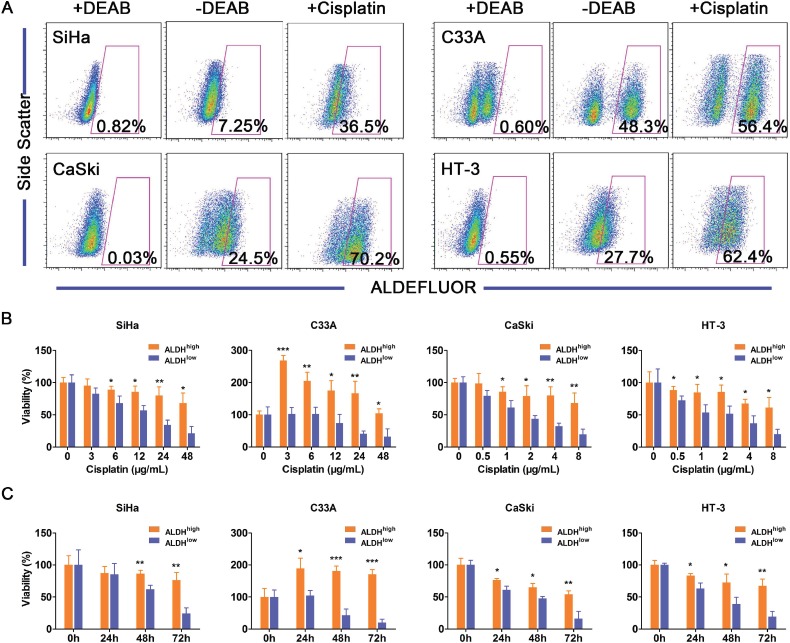
ALDH^high^ cells are more resistant to cisplatin than ALDH^low^ cells A, The ALDH activity of the cervical cancer cell lines was analyzed after exposure to cisplatin (3 µg/mL for SiHa and C33A cells or 1 µg/mL for CaSki and HT-3 cells) for 3 days. The percentage of ALDH^high^ cells in the 4 cervical cancer cell lines was analyzed by flow cytometry following exposure to cisplatin for 3 days. B, Cell viability of the ALDH^high^ and ALDH^low^ cervical cancer cells was measured using an MTT assay after treatment with different concentrations of cisplatin for 24 h. C, Cell viability of the ALDH^high^ and the ALDH^low^ cervical cancer cells was measured using an MTT assay after treatment with a constant dose of cisplatin for 0, 24, 48 or 72 h. ^*^, *p*<0.05; ^**^, *p*<0.01; ^***^, *p*<0.001. Data represent mean ± SD of triplicate experiments.

The resistance of CSCs to current chemotherapeutics is thought to be responsible for cancer recurrence and metastasis [46]. Because cisplatin is one of the most commonly used chemotherapeutic drugs in the treatment of cervical cancer, we tested the effects of cisplatin on ALDH^high^ and ALDH^low^ cervical cancer cells. After treatment with cisplatin, the population of ALDH^high^ cells expanded from 7.25% to 36.5% in SiHa cells, 48.3% to 56.4% in C33A cells, 24.5% to 70.2% in CaSki cells and 27.7% to 62.4% in HT-3 cells (Figure 5A). These data suggested that the ALDH^high^ cells, but not the ALDH^low^ cervical cancer cells, are resistant to cisplatin treatment.

Furthermore, ALDH^high^ and ALDH^low^ cells isolated from 4 cervical cancer cell lines were exposed to different concentrations of cisplatin for 24 h, and cell viability was determined using an MTT assay. Cisplatin caused dose-dependent decreases in the viability of both the ALDH^high^ and the ALDH^low^ cervical cancer cells (Figure 5B). ALDH^high^ SiHa cells were significantly more resistant to cisplatin concentrations of ≥6 µg/mL than the ALDH^low^ SiHa cells. ALDH^high^ CaSki cells were significantly more resistant to ≥1 µg/mL cisplatin than the ALDH^low^ CaSki cells. ALDH^high^ HT-3 cells were significantly more resistant to concentrations of ≥0.5 µg/mL cisplatin than the ALDH^low^ HT-3 cells. These results indicated that ALDH^high^ cells are more resistant to cisplatin than ALDH^low^ cells when exposed to the proper concentration for a limited period of time. The viability of the ALDH^high^ C33A cells was significantly greater than that of the ALDH^low^ cells after exposure to any concentration of cisplatin. Cisplatin caused a dose-dependent decrease in the viability of the ALDH^low^ C33A cells. Surprisingly, the viability of the ALDH^high^ C33A cells was significantly enhanced after exposure to cisplatin compared with cells that had not been treated with cisplatin.

Cell viability was also determined by the MTT assay after exposure to constant concentration of cisplatin for 24, 48, or 72 h (Figure 5C). Cisplatin caused a time-dependent decrease in the viability of both ALDH^high^ and ALDH^low^ cells from the SiHa, CaSki and HT-3 cells. ALDH^high^ SiHa cells were significantly more resistant to ≥48 h of treatment with cisplatin than ALDH^low^ cells. In the CaSki and HT-3 cells, ALDH^high^ cells were significantly more resistant than ALDH^low^ cells to cisplatin treatment for ≥24 h. The results from these 3 cell lines indicate that ALDH^high^ cells are more resistant to constant concentration of cisplatin than ALDH^low^ cells for certain periods of time. The viability of ALDH^high^ C33A cells was significantly greater than that of ALDH^low^ cells after exposure to cisplatin. Cisplatin caused a time-dependent decrease in the viability of ALDH^low^ C33A cells, while the viability of ALDH^high^ C33A cells was significantly enhanced following exposure to cisplatin compared to cells that had not been treated with cisplatin.

In summary, these results suggest that ALDH^high^ cervical cancer cells are more resistant to chemotherapy than ALDH^low^ cells.

### ALDH^high^ cells express high levels of stem cell-associated markers

**Figure 6:**
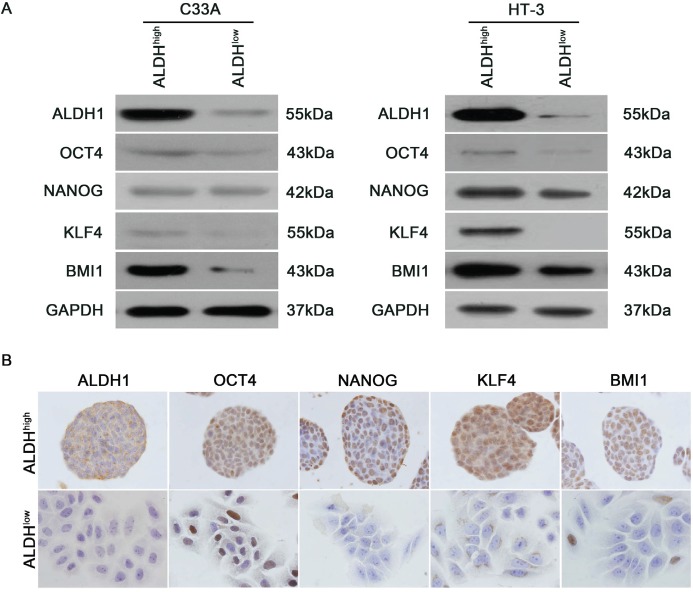
Expression of stem cell-associated markers in ALDH^high^ and ALDH^low^ cells A, Western blot analysis of the protein levels of the stem cell-associated transcription factors OCT4, NANOG, KLF4 and BMI1 in the ALDHhigh and ALDHlow subpopulations from C33A and HT-3 cells. GAPDH was used as a loading control. B, The expression of stem cell-associated transcription factors in tumorspheres formed by ALDHhigh and ALDHlow SiHa cells was measured by IHC analysis.

Stem cell-related transcription factors are important for maintaining the self-renewal of embryonic stem cells. To clarify whether the ALDH^high^ cervical cancer cells express stem cell-related transcription factors, western blot analysis was performed to assess the expression of OCT4, NANOG, KLF4 and BMI1 in ALDH^high^ and ALDH^low^ cells. ALDH^high^ C33A and HT-3 cells were found to express higher levels of OCT4, NANOG, KLF4 and BMI1 than ALDH^low^ cells (Figure 6A). IHC analysis was also performed on the tumorspheres formed by the ALDH^high^ and ALDH^low^ SiHa cells to evaluate the expression of stem cell-associated markers (Figure 6B). Similar to the western blot analysis, the stem cell-associated transcription factors OCT4, NANOG, KLF4 and BMI1 were detected in the tumorspheres formed by the ALDH^high^ SiHa cells but not the ALDH^low^ SiHa cells. These data indicate that ALDH^high^ cervical cancer cells display a nuclear stemness signature.

### ALDH^high^ cells from primary cervical cancers possess CSC characteristics

Our data suggest that ALDH^high^ cells from cervical cancer cell lines display characteristics of CSCs. However, whether ALDH^high^ cells derived from primary cervical cancers possess the same characteristics remained unknown. To address this question, primary cervical cancer tissues were processed into single cell suspensions and injected subcutaneously into NOD/SCID mice to create tumor xenografts. Only 5 out of 28 primary cervical cancer specimens were successfully serially transplanted for 5 generations; the estimated engraftment rate was 18%. The generated xenografts were resected and dissociated into single cell suspensions. Cells were plated in 24-well plates at a density of 10^3^ cells/well and cultured for tumorsphere formation. All tumorspheres generated from the same cervical cancer tissue were collected, digested into single cell suspensions, labeled with the ALDEFLUOR kit, analyzed and sorted by FACS.

**Figure 7:**
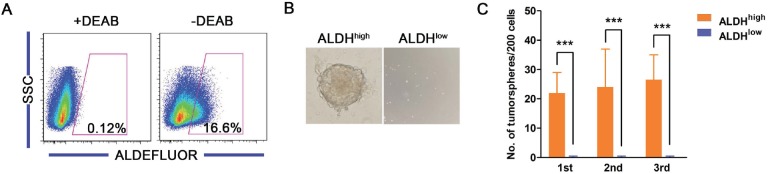
Characteristics of ALDH^high^ cells from primary cervical cancers A, The ALDH enzyme activity of primary cervical cancer cells was analyzed by flow cytometry. B, Representative photos of tumorspheres formed by ALDH^high^ and ALDH^low^ cells sorted from primary cervical cancer are shown. C, The number of tumorspheres formed by ALDH^high^ and ALDH^low^ cells isolated from the primary cervical cancer was counted from 3 consecutive passages. ^***^, *p*<0.001. Data represent mean ± SD of triplicate experiments.

A subpopulation of ALDH^high^ cells (11-23%) was detected in all 5 of the primary tumors tested (Figure 7A). The 10% of the cell population with the highest and the lowest ALDH activity were sorted as the ALDH^high^ and the ALDH^low^ cells, respectively, for the subsequent experiments. As shown in Figure 7B, the ALDH^high^, but not the ALDH^low^, primary cervical cancer cells were capable of generating tumorspheres in suspension culture. ALDH^high^ cells formed tumorspheres with a frequency of approximately 10% in 3 consecutive passages (Figure 7C), while ALDH^low^ cells did not generate tumorspheres (but did generate some cell aggregates). These data suggest that the ALDH^high^ primary cervical cancer cells have the ability to self-renew.

To study the tumor formation capacity of the ALDH^high^ and the ALDH^low^ primary cervical cancer cells, 10^6^, 10^5^, 10^4^ or 10^3^ cells were subcutaneously injected into NOD/SCID mice, and the mice were monitored for tumor growth for 20 weeks. As summarized in Table 2, the ALDH^high^ primary cervical cancer cells exhibited enhanced tumorigenicity compared with the ALDH^low^ cells. Among the 5 primary tumor samples, the tumor-initiating frequency of the ALDH^high^ cells was as high as 6-200 times that of the ALDH^low^ cells (Table 2). Furthermore, the tumors generated from ALDH^high^ cells were significantly larger and grew faster than the tumors derived from their ALDH^low^ counterparts. These results indicate that ALDH activity may be a functional marker of CSCs in primary cervical cancer.

**Table 2:** Tumorigenic capacity of ALDH^high^ and ALDH^low^ cells in NOD/SCID mice from primary tumor of cervix

Primary tumor	Sub-population	Cell dose				Tumor-Initiating frequency (95% Interval)	p value
		10^6^	10^5^	10^4^	10^3^		
PT1	ALDH^high^	5/5	4/5	3/5	1/5	1:30,680(1:85,460—1:11,014)	<0.001
	ALDH^low^	3/5	1/5	1/5	0/5	1:733,639(1:1,978,007—1:272,105)	
PT2	ALDH^high^	--	5/5	2/5	0/5	1:20,275(1:62,492—1:6,579)	<0.001
	ALDH^low^	2/5	1/5	0/5	--	1:1,462 107(1:4,770,331—1:448,136)	
PT3	ALDH^high^	--	5/5	2/5	1/5	1:14,294(1:44,503—1:4,591)	0.012
	ALDH^low^	5/5	3/5	1/5	0/5	1:93,382(1:263,758—1:33,062)	
PT4	ALDH^high^	--	5/5	4/5	3/5	1:3,596(1:9,879—1:1,309)	<0.001
	ALDH^low^	3/5	2/5	0/5	--	1:722,444(1:1,945,037—1:268,337)	
PT5	ALDH^high^	4/5	2/5	0/5	--	1:462,760(1:1,216,059—1:176,099)	0.008
	ALDH^low^	1/5	0/5	--	--	1:4,983,289(1:35,191,497—1:705,659)	

## DISCUSSION

Several approaches have been utilized to identify CSCs from various human malignancies, including cell surface markers, side population phenotype, spheres formation and ALDH activity assays [21]. ALDH activity (measured using the ALDEFLUOR assay) was first used to isolate leukemia stem cells [28]. Subsequently, ALDH activity has been successfully used as a CSC marker for many solid tumors, including breast [29], colon [30, 31], bladder [33], prostate [34], lung [36], head and neck [38], endometrium [39], ovary [40] and thyroid [47]. Therefore, ALDH activity may have potential as a promising universal marker for the identification and isolation of stem cells from various solid tumors. However, previous reports have not indicated whether ALDH activity can also be used as a CSC marker in cervical cancer.

In the present study, the ALDH^high^ cells isolated from 4 cervical cancer cell lines and 5 primary cervical cancer xenografts were demonstrated to fulfill the functional criteria for CSCs. Firstly, using the tumorsphere formation assay, ALDH^high^ cervical cancer cells were found to have self-renewal capacity. ALDH^high^ cells formed significantly more tumorspheres in both single cell culture and low density cell culture than ALDH^low^ cells. Furthermore, ALDH^high^ cells formed more tumorspheres in single cell culture than in low density cell culture (Figure 2B and 2C). Similar results have been reported for mammospheres, and cell aggregation in the low density cell cultures might contribute to the decreased formation of tumorspheres [29]. Secondly, ALDH^high^ cervical cancer cells could differentiate and reconstitute the cellular hierarchy *in vitro* and *in vivo*. After 2 weeks of culture in medium containing FBS, ALDH^high^ cells differentiated into many ALDH^low^ cells, while ALDH^low^ cells generated few ALDH^high^ cells and predominantly maintained the ALDH^low^ phenotype (Figure 4A-4D). The ALDH1-positive population could be detected in all of the tumor xenograft tissues formed by the ALDH^high^ cells, but no ALDH1-positive cells were found in the tumors formed by the ALDH^low^ cells (Figure 4E). Therefore, only the ALDH^high^ cells, not the ALDH^low^ cells, could differentiate and re-establish the cellular hierarchy *in vitro* and *in vivo*. Thirdly, ALDH^high^ cervical cancer cells had tumor initiating capacity *in vivo*. The tumors formed by the ALDH^high^ cells were larger and grew faster than those derived from the ALDH^low^ cells (Figure 3A). A shorter tumor-free period and a lower tumor-free rate were observed in mice injected with the ALDH^high^ cells than in mice injected with ALDH^low^ cells (Figure 3B). The tumor-initiating frequency of the ALDH^high^ cells was significantly higher than that of the ALDH^low^ cells (Table 1). Taken together, these data indicate that the ALDH^high^ cells are indeed CSCs in cervical cancer, similar to the results of previous ALDH studies in other solid tumors [29, 30, 33, 34, 36, 41].

ALDH expression and activity has been reported to be significantly higher in taxane- and platinum-resistant ovarian cancer cell lines [40]. Rahadiani et al. have reported that ALDH^high^ endometrioid adenocarcinoma cells are more resistant to cisplatin treatment than ALDH^low^ cells [39]. In this study, the ALDH^high^ cells were more resistant to cisplatin treatment than the ALDH^low^ cells (Figure 5), suggesting that ALDH^high^ CCSCs exhibit chemoresistance similar to the CSCs found in other solid tumors [39, 48]. Surprisingly, the viability of the ALDH^high^ C33A cells was significantly enhanced following exposure to cisplatin (Figure 5B and C). We attribute this to stimulated proliferation or enhanced activity of succinic acid dehydrogenase, which is the enzyme activity measured by the MTT assay. Further experiments are necessary to clarify the mechanisms that underlie the enhanced viability of the ALDH^high^ C33A cells.

The direct isolation of CSCs from uncultured human primary colon [30] and prostate [34] cancer cells and sorting based on ALDH activity has been reported. ALDH^high^ CSCs in breast cancer [29] and pancreatic cancer [37] have been successfully isolated from primary cancer xenografts established in mice. In the present study, uncultured primary cervical cancer cells were first used to investigate the tumorigenicity of the ALDH^high^ and the ALDH^low^ cells. Unfortunately, neither the ALDH^high^ nor the ALDH^low^ cells from primary cervical cancer resulted in tumors in NOD/SCID mice, similar as the report about ovarian cancer [40]. Three possibilities may contribute to the failure of CSC isolation directly from primary uncultured cervical cancer cells: (1) ALDH-positive non-cancerous cells, including white blood cells, stromal cells or normal stem cells, were abundant in the primary cancer tissues and contaminated the cancerous cells during tumor formation; (2) all primary cervical cancer tissues obtained from surgery are likely below the clinical stage of II a, and most of these cancerous cells may be too fragile to be sorted; or (3) the cells may be not resilient enough to form tumors in NOD/SCID mice. In the present study, CSCs were isolated from 5 generations of cervical cancer serially xenografted in NOD/SCID mice but not from uncultured primary cervical cancer cells. The following 3 possibilities may explain this phenomenon: (1) After more than 5 serial xenograft generations in NOD/SCID mice, primary cervical cancer cells acquired the capacity to form tumors; this idea is consistent with the notion that cancer cells can acquire enhanced tumorigenicity through serial transplantation in mice. (2) In this study, only 5 of the 28 cases of primary cervical cancer (approximately 18%) could successfully be serially transplanted for more than 5 generations in mice, indicating that the cancer cells in most primary cancer tissues were too fragile to form tumors in NOD/SCID mice. (3) The cells used for the tumor formation assay were isolated from tumorspheres, which decreases the likelihood of contamination with ALDH-positive non-cancerous cells from the primary cancer tissues.

ALDH1 has been reported to be a marker for normal mammary stem cells [29]. In this study, ALDH1 expression was found in the basal cells of normal cervical tissue (Figure 1A, upper panel). The basal cells were recognized to contain cervical stem cells. Therefore, ALDH1 may be a marker of normal cervical stem cells. Further experiments should be designed to isolate and test the ALDH1-positive cervical basal cells to verify whether ALDH1 can be used as a marker of normal cervical stem cells.

In summary, this report is the first to describe the use of high ALDH activity to isolate CSCs from cervical cancer cell lines and primary cervical cancer cells. These ALDH^high^ cervical cancer cells possess the ability to self-renew and differentiate and have enhanced tumorigenicity. Additionally, these cells exhibit chemoresistance and express high levels of stem cell-related transcription factors. Based on this study, ALDH activity may be used as a cytoplasmic marker for CCSCs, and a target to explore novel strategies for diagnosis, prognosis and therapy.

## METHODS

### Ethics Statement

Investigation has been conducted in accordance with the ethical standards and according to the Declaration of Helsinki and according to national and international guidelines and has been approved by the review board of the First Affiliated Hospital of Xi'an Jiaotong University.

### Cell lines and culture conditions

The human cervical cancer cell lines SiHa, C33A, CaSki and HT-3 were obtained from the American Type Culture Collection (ATCC; Manassas, VA). SiHa and C33A cells were cultured in Dulbecco's Modified Eagle Medium-high glucose (DMEM; Sigma-Aldrich, St. Louis, MO) supplemented with 10% fetal bovine serum (FBS; Invitrogen, Carlsbad, CA). CaSki and HT-3 cells were cultured in RPMI-1640 (Sigma-Aldrich) and McCoy's 5A medium (Sigma-Aldrich), respectively, supplemented with 10% FBS. All cell lines were maintained at 37°C in an atmosphere containing 5% carbon dioxide.

### Human tissue specimens, primary cervical cancer tissue processing and xenograft lines

A total of 17 normal cervical tissues and 53 cervical cancer tissues were obtained from the First Affiliated Hospital of Xi'an Jiaotong University. The procedures followed approved medical ethics practices, and the patients provided their informed consent before the specimens were collected. Fresh cervical cancer tissues were obtained from 28 patients after radical hysterectomy and used for xenograft experiments. Single cell suspension was generated by mincing and digesting the tissue with 100 U/mL collagenase IV (GIBCO, Grand Island, NY) in basal medium at 37°C overnight. Xenograft lines were established by subcutaneous implantation of the primary cervical cancer cells in 6- to 8-week old NOD/SCID mice (Charles River Laboratories, Wilmington, MA). Once established, the solid tumor xenografts were serially passaged using the same technique.

### Immunohistochemistry (IHC)

Formalin-fixed and paraffin-embedded tissue specimens were sliced into 4 mm sections, which were then deparaffinized and hydrated. An endogenous antigen retrieval procedure was performed using citric acid buffer (10 mmol/L citrate buffer, pH 6.0). The slides were incubated with a mouse monoclonal antibody raised against human ALDH1 (BD Biosciences, Franklin Lakes, NJ) or Ki67 (Santa Cruz, CA) overnight at 4°C, then with secondary antibodies for 30 min at room temperature, followed by diaminobenzidine development. All slides were examined under an Olympus-CX31 microscope (Olympus, Tokyo, Japan).

### Flow cytometry analysis and FACS isolation of cells

The ALDH enzymatic activity of the cells was measured using the ALDEFLUOR kit (Stem Cell Technologies, Vancouver, BC, Canada), according to the manufacturer's instructions. The brightly fluorescent ALDH-expressing cells were detected using a FACSCalibur or FACSAria flow cytometer (BD Biosciences). As a negative control, cells were stained under identical conditions after treatment with the specific ALDH inhibitor diethylaminobenzaldehyde (DEAB). The data were analyzed using FlowJo software (Tree Star Inc., Ashland, USA). For FACS, the cells were labeled using the ALDEFLUOR kit and sorted using a FACSAria cell sorter (BD Biosciences).

### Tumorsphere culture

Cells were maintained in stem cell media consisting of DMEM/F12 basal media, N2 and B27 supplements (Invitrogen), 20 ng/mL human recombinant epidermal growth factor (EGF) and 20 ng/mL basic fibroblastic growth factor (bFGF; PeproTech Inc., Rocky Hill, NJ). For the tumorsphere formation assay, cells were plated at a density of 200 cells/well in 24-well ultra-low attachment plates or at a density of 1 cell/well in 96-well plates and maintained in stem cell medium. Tumorspheres that arose within 2 weeks were recorded. For serial tumorsphere formation assays, the spheres were harvested, disaggregated with 0.25% trypsin/EDTA, filtered through a 40 µm mesh and re-plated as described above. For each cell type, triplicate samples were done and the spheres were counted by two individuals in a blind fashion.

### Western blot

Cells were lysed in a lysis buffer (50 mM Tris-HCl, pH 7.4; 150 mM NaCl; 2 mM EDTA; 1% NP-40; and 0.1% sodium dodecyl sulfate) that contained a protease inhibitor cocktail (Complete Mini; Roche Diagnostics, Branchburg, NJ). The membranes were incubated with antibodies raised against ALDH1 (BD Biosciences), BMI1 (Millipore, Billerica, Mass), OCT4 (Santa Cruz), KLF4 (Santa Cruz) and GAPDH (Santa Cruz) at 4°C overnight, followed by a secondary incubation with horseradish peroxidase-conjugated immunoglobulin G (IgG; Thermo Fisher Scientific, New York, NY). The membranes were briefly incubated with an enhanced chemiluminescence reagent (Millipore, Billerica, Mass), then visualized on x-ray films.

### Drug resistance and MTT assay

For drug resistance assays, cells were plated in 96-well plates at a density of 10^4^ cells/well and allowed to recover overnight before initiating drug treatments. The cells were exposed to various concentrations of cisplatin (0, 3, 6, 12, 24, or 48 µg/mL for SiHa and C33A cells or 0, 0.5, 1, 2, 4, or 8µg/mL for CaSki and HT-3 cells) for 24 h, and the cell viability was measured. In separate experiments, the cells were exposed to a constant concentration of cisplatin (3 µg/mL for SiHa and C33A cells or 1 µg/mL for CaSki and HT-3 cells) for 24, 48 or 72 h, and the cell viability was measured.

Cell viability was assessed using a 3-(4, 5-Dimethyl-1, 3-thiazol-2-yl)-2, 5-diphenyl-2H-tetrazol-3-ium bromide (MTT; Sigma-Aldrich) assay. Following the manufacturer's instructions, 20 µL of MTT solution were added to 200 µL of the culture media. The plates were then incubated for 4 h at 37°C, and the optical density was measured at 490 nm.

### *In vivo* tumor formation assays

The ALDH^high^ and ALDH^low^ cells were sorted, re-suspended in 200 µL of 1:1 PBS/Matrigel (BD Biosciences) and injected subcutaneously into the flanks of 6- to 8-wk old female NOD/SCID mice; the left flank of the mouse received the ALDH^high^ cells, whereas the right flank received the ALDH^low^ cells. Engrafted mice were inspected twice per week by visual observation and palpation for the appearance of tumors. The tumor volume (V) was determined from the length (a) and the width (b) of the tumor, using the formula V=ab^2^/2 [49]. A portion of each tumor tissue was fixed in 10% formaldehyde and embedded in paraffin for IHC analysis. The frequency of tumorigenic cells (estimated with upper–lower limits) was calculated by limiting-dilution analysis [50].

### Statistical analysis

Statistical analyses were performed using GraphPad Prism 5.01 software (La Jolla, CA, USA). In comparisons of 2 groups, Student's *t*-test was used to determine the statistical significance. To examine differences among 3 groups, an ANOVA analysis was performed. Kaplan-Meier survival analysis was performed and survival curve comparison analyses were performed using the log-rank (Mantel-Cox) test. P values of ≤0.05 were regarded as statistically significant.

## ACKNOWLEDGEMENTS

This research was supported by a grant for Distinguished Young Scientists (No. 30725043) from the National Natural Science Foundation of China.
